# The Prevalence of Metabolic Dysfunction-Associated Fatty Liver Disease and Its Association with Physical Function and Prognosis in Patients with Acute Coronary Syndrome

**DOI:** 10.3390/jcm11071847

**Published:** 2022-03-26

**Authors:** Takumi Noda, Kentaro Kamiya, Nobuaki Hamazaki, Kohei Nozaki, Takafumi Ichikawa, Masashi Yamashita, Shota Uchida, Emi Maekawa, Tasuku Terada, Jennifer L. Reed, Minako Yamaoka-Tojo, Atsuhiko Matsunaga, Junya Ako

**Affiliations:** 1 Department of Rehabilitation Sciences, Kitasato University Graduate School of Medical Sciences, Sagamihara 252-0373, Japan; ap15328@st.kitasato-u.ac.jp (T.N.); ap12344@st.kitasato-u.ac.jp (M.Y.); ap14308@st.kitasato-u.ac.jp (S.U.); myamaoka@med.kitasato-u.ac.jp (M.Y.-T.); atsuhikonet@gmail.com (A.M.); 2 Department of Rehabilitation, Kitasato University School of Allied Health Sciences, Sagamihara 252-0373, Japan; 3 Department of Rehabilitation, Kitasato University Hospital, Sagamihara 252-0329, Japan; hamanobu0317@gmail.com (N.H.); 0818.n.kohei@gmail.com (K.N.); takafumi@kitasato-u.ac.jp (T.I.); 4 Department of Cardiovascular Medicine, Kitasato University School of Medicine, Sagamihara 252-0374, Japan; emimae1207@med.kitasato-u.ac.jp (E.M.); jako@kitasato-u.ac.jp (J.A.); 5 Exercise Physiology and Cardiovascular Health Lab, Division of Cardiac Prevention and Rehabilitation, University of Ottawa Heart Institute, Ottawa, ON K1Y 4W7, Canada; tterada@ottawaheart.ca (T.T.); jreed@ottawaheart.ca (J.L.R.); 6 School of Epidemiology and Public Health, Faculty of Medicine, University of Ottawa, Ottawa, ON K1N 6N5, Canada; 7 School of Human Kinetics, Faculty of Health Sciences, University of Ottawa, Ottawa, ON K1N 6N5, Canada

**Keywords:** metabolic dysfunction-associated fatty liver disease, acute coronary syndrome, liver dysfunction, frailty, sarcopenia, physical function

## Abstract

It is believed that patients with acute coronary syndrome (ACS) are at an increased risk of nonalcoholic fatty liver disease (NAFLD), which can lead to sarcopenia and physical dysfunction. However, the relationship between metabolic dysfunction-associated fatty liver disease (MAFLD) and physical dysfunction and prognosis remains unclear. We investigated the prevalence of MAFLD in patients with ACS to assess the relationship between MAFLD and muscle strength, walking speed, and 6-min walking distance (6 MWD). We reviewed patients with ACS who were assessed for hepatic steatosis using the fatty liver index, and the results were further assessed to determine the presence of MAFLD. Among 479 enrolled hospitalized patients, MAFLD was identified in 234 (48.9%) patients. Multiple regression analysis revealed that MAFLD was independently associated with lower leg strength, gait speed, and 6 MWD (leg strength, *p* = 0.020; gait speed, *p* = 0.003 and 6 MWD, *p* = 0.011). Furthermore, in multivariate Poisson regression models after adjustment for clinical confounding factors, combined MAFLD and reduced physical functions were significantly associated with a higher incidence of clinical events. MAFLD is common in hospitalized patients with ACS and is associated with impaired physical function. Also, the coexistence of MAFLD and lower physical function predict the incidence of clinical events in patients with ACS.

## 1. Introduction

Nonalcoholic fatty liver disease (NAFLD) is recognized as the most prevalent chronic liver disease worldwide, with an estimated global prevalence of 20–30% [1,2,3]. NAFLD is a multisystem disease that affects organs and regulatory pathways other than the liver [4,5,6,7]. Liver fibrosis caused by NAFLD progression is associated with increased mortality [8,9]. Furthermore, NAFLD is more common in patients with cardiovascular diseases (CVD) [2,5,10,11], including acute coronary syndrome (ACS), thereby increasing the risk of recurring CVD events. Therefore, the relationship between NAFLD and cardiovascular events has attracted attention for CVD prevention [3,10]. Recently, a new name and definition for NAFLD have been proposed to better understand fatty liver [12]; this new name is metabolic dysfunction-associated fatty liver disease (MAFLD). The newly proposed definition is based on the coexistence of hepatic fat deposition and overweight/obesity, diabetes mellitus (DM), or multiple metabolic abnormalities and can be diagnosed regardless of daily alcohol consumption or other liver diseases [12]. Therefore, it has been proposed that MAFLD is a more appropriate term to describe liver disease with a background of metabolic abnormalities. Metabolic diseases, such as obesity and DM, are the risk factors for developing ACS [13,14], and patients with ischemic heart disease (IHD) often present with several metabolic risk factors. Thus, the prevalence of MAFLD in patients with ACS may be higher than in the general population due to the combined presence of metabolic risk factors such as DM and obesity in this population despite an increased prevalence of these risk factors in the general population too.

MAFLD in patients with ACS is more likely to result in decreased physical function. Previous studies have demonstrated that NAFLD in patients with CVD has often been associated with muscle weakness or reduced walking speed due to insulin resistance and chronic inflammation [15]. The combination of NAFLD and physical dysfunction is associated with a poor prognosis and decreased quality of life (QOL) [16,17,18]. However, it is unclear whether MAFLD in patients with ACS is involved in the decline in physical functions such as lower leg strength and walking speed. Thus, this study aimed to investigate the prevalence of MAFLD in patients with ACS and to clarify the relationship between MAFLD and physical dysfunction. In addition, we examined the prognostic relevance of combined MAFLD and physical dysfunction in patients with ACS.

## 2. Materials and Methods

### 2.1. Study Population

A total of 479 patients with ACS admitted to the Kitasato University Hospital Cardiovascular Center between May 2008 and November 2018 were included in this single-center retrospective observational study. All the patients had acute ST-segment elevation, non-ST-segment elevation myocardial infarction, or unstable angina diagnosed according to the American College of Cardiology/American Heart Association guidelines [19] and were assessed for the presence of hepatic steatosis by fatty liver index (FLI). Patients undergoing maintenance hemodialysis were excluded from the study. The study protocol was designed according to the tenets of the Declaration of Helsinki and approved by the Ethics Committee of the Kitasato University Medical Ethics Organization (KMEO) (no. KMEO B18-075). Since the present study is an observational study that did not involve invasive procedures or interventions, written informed consent was not required according to the principles set forth in the “Ethical Guidelines for Medical and Health Research for Subjects” by the Japanese Ministry of Health, Labor and Welfare. Therefore, informed consent was waived by the KMEO according to the institutional guidelines for retrospective observational studies. All participants were informed that they could choose to withdraw at any point or refuse to participate in the study.

### 2.2. Data Collection and Assessment of MAFLD

The clinical characteristics of the patients (age, sex, body mass index (BMI), underlying disease (angina pectoris or myocardial infarction), laboratory examination results, vital signs (blood pressure and heart rate), echocardiographic data, etc.) were collected from the electronic medical records at the time of discharge. Additionally, the clinical information (comorbidities and medication use) at discharge was recorded. All-cause death and emergency CVD re-hospitalization was the endpoint of this study, and the time to endpoint was calculated as the number of days from the date of discharge to the event’s occurrence. Follow-up was performed after discharge, and the last day of the period was recorded as the censoring date for all-cause mortality and emergency hospitalization events for CVD.

We assessed the presence of hepatic steatosis in each patient using the FLI [20,21]. The FLI is an algorithm based on waist circumference (WC), BMI, triglyceride (TG), and gamma-glutamyl transpeptidase (γ-GTP) for the prediction of fatty liver, and is easy to employ since the individual components are routinely measured in clinical practice. It has been validated as a practical and reliable technique to diagnose NAFLD in large epidemiology studies. The FLI was calculated using the formula FLI = e^0.953 × ln [TG] + 0.139 × BMI + 0.718 × ln [^^γ^^-GTP] + 0.053 × WC − 15.745^/(1 + e^0.953 × ln [TG] + 0.139 × BMI + 0.718 × ln [^^γ^^-GTP] + 0.053 × WC − 15.745^) × 100. The units for TG, γ-GTP, and WC were mg/dL, U/L, and cm, respectively. Hepatic steatosis was defined as FLI ≥ 35 for men and FLI ≥ 20 for women [4].

MAFLD was diagnosed based on FLI-confirmed hepatic steatosis with any of the three metabolic conditions: DM, overweight/obesity, or at least two metabolic risk abnormalities. According to the MAFLD definition [12], metabolic risk abnormalities were defined as the presence of the following criteria: (1) WC ≥ 90 cm in men and 80 cm in women, (2) prediabetes (hemoglobinA1c 5.7 to 6.4%), (3) blood pressure ≥ 130/85 mmHg or under antihypertensive therapy, (4) high-density lipoprotein cholesterol (HDL-C) < 40 mg/dL in men and <50 mg/dL in women, (5) TG ≥ 150 mg/dL or specific drug treatment, and (6) C-reactive protein (CRP) level > 2 mg/L. The homeostasis model assessment-insulin resistance (HOMA-IR) score was not investigated in this study.

### 2.3. Physical Function Tests

Physical function tests such as leg strength, gait speed, and 6-min walking distance (6 MWD) were measured before discharge. Leg strength was defined as the maximal isometric quadriceps muscle strength and was measured using a portable dynamometer (µ-Tas; ANIMA, Tokyo, Japan). The detailed measurement method and reliability are described in our previous study (AJM). Briefly, the patient was seated in a chair, and the maximum isometric voluntary contraction of the quadriceps muscle was measured for 5 s, twice in each leg with the hip joint flexed approximately at 90° using a non-extensible strap connected to a strain gauge at the ankle. Measurements were performed on the right and left quadriceps in succession. Left and right maximal muscle strength values were averaged and expressed as absolute values (kg) and relative to body mass (%BM).

Usual gait speed was measured when the participants were asked to walk for 10 m in the middle of a 16 m sidewalk at their average pace. The normal walking rate of each participant was calculated by dividing the distance (m) by the time (s).

The 6 MWD was measured under the supervision of a technician according to the guidelines of the American Thoracic Society (ATS) [22]. Patients were instructed to walk at their own pace along a straight, flat corridor marked at 1 m intervals, and the distance (in m) was recorded after 6 min.

### 2.4. Statistical Analysis

The results for distributed continuous data are presented as medians (interquartile range (IQR)). Categorical variables were expressed as numbers and percentages. The patients were divided into two groups: those with MAFLD and those without MAFLD. The baseline characteristics were compared using the Mann-Whitney U test for continuous variables and the Chi-square test for categorical variables, as appropriate. Non-normally distributed variables were transformed to a logarithmic scale for analysis. Multiple imputations using R with the “mice” package version 3.13.0 [23] generated 20 datasets with complemented missing values.

We used the Cochran-Armitage trend test to analyze the relationship between the prevalence of MAFLD and age or DM. We also used the Euler diagrams (area-proportional diagrams) to visualize the number of patients in the disjoint and overlapping areas of the three metabolic conditions domains using R with the “eulerr” package version 6.1.0 (https://cran.r-project.org/package=eulerr (accessed on 1 September 2021)).

To evaluate the strength of the association between leg strength, gait speed, 6 MWD, and MAFLD, we used multiple linear regression models adjusted for the following variables: age, sex, BMI, log maximum creatine kinase (CK), number of diseased vessels, prior heart failure, hypertension, dyslipidemia, DM, smoking, log albumin, and log hemoglobin. In addition, multiple linear regression analysis adjusted for the same variables was performed to assess the association of MAFLD with leg strength, gait speed, and 6 MWD when stratified by the total number of the three metabolic conditions, that is, DM, overweight/obesity, or metabolic risk abnormalities.

We divided the subject into four groups, the non-MAFLD/high physical function, non-MAFLD/low physical function, MAFLD/high physical function, and MAFLD/low physical function groups. To examine the association between the prognosis of combined MAFLD and physical dysfunction, we performed Poisson regression analysis after adjusting for age, sex, BMI, log maximum CK, the number of diseased vessels, prior heart failure, hypertension, dyslipidemia, DM, smoking, log albumin, and log hemoglobin. We estimated the incidence rate ratio (IRR) and 95% confidence interval (CI). Based on previous studies [24,25], the cutoff values for lower leg muscle strength, gait speed, or 6MWD reduction were ≤35% BM, <1.0 m/s, and <400 m, respectively. The endpoint was the composite outcome of all-cause death and CVD re-hospitalization.

All statistical analyses were performed using the R Studio statistical software (version 3.6.2; R: A language and environment for statistical computing, R Core Team, R Foundation for Statistical Computing, Vienna, Austria, 2019, https://www.R-project.org (accessed on 11 March 2022)). The level of statistical significance was set at *p* < 0.05. 

## 3. Results

The baseline characteristics of the study participants are shown in Table 1. The median age of the study population was 65 years, with 80.8% men and 19.2% women. The median lower extremity muscle strength, gait speed, and 6 MWD of the study participants were 47.4% BM, 1.2 m/s, and 486 m, respectively.

**Figure 1:**
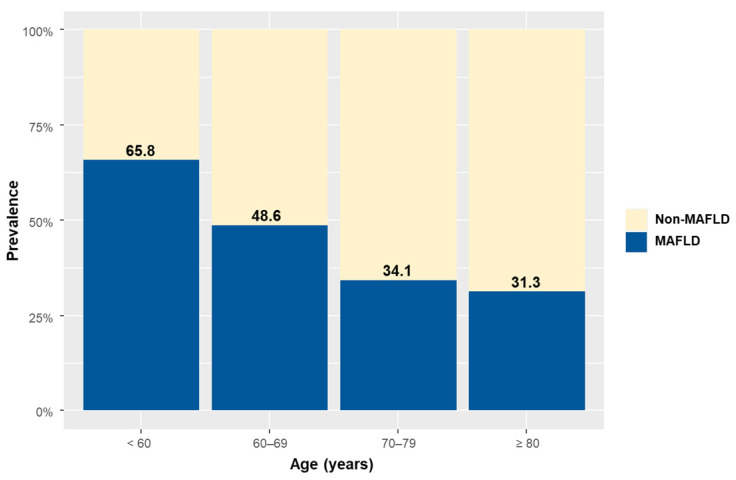
Prevalence of metabolic dysfunction-associated fatty liver disease according to age category.

According to the predefined definition, 234 patients (48.9%) had MAFLD. As shown in Figure 1, MAFLD was more common in younger patients and was found in 65.8%, 48.6%, 34.1%, and 31.3% of patients under 60 years, in their 60s, in their 70s, and 80 years and older, respectively (*p* for trend < 0.001). The prevalence of MAFLD in the two groups of patients without or with DM was 44.4% and 52.7%, respectively (*p* for trend = 0.069) (Supplementary Figure S1).

**Figure 2:**
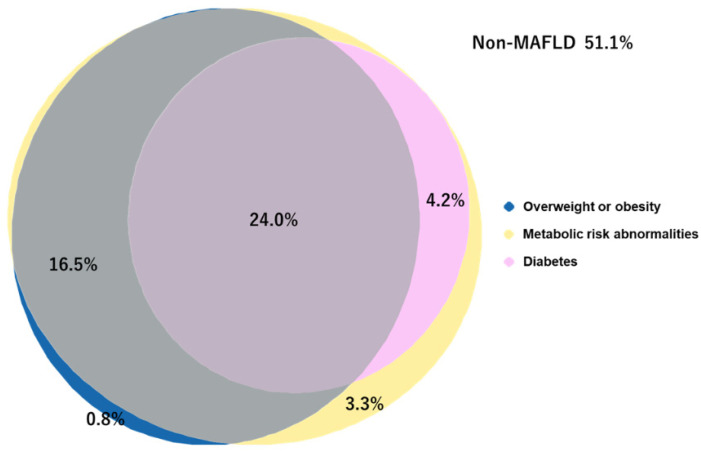
The proportion of overlap and non-overlap among metabolic conditions in patients with metabolic dysfunction-associated fatty liver disease (MAFLD). Euler diagrams (area-proportional diagrams) visualize the number of patients in the disjoint and overlapping three metabolic disorders. Three circles indicate the prevalence of overweight or obesity, metabolic risk abnormalities, and diabetes, respectively. The percentage of the population in the single and overlapped domains is also shown. The rate is about the total cohort (*n* = 479).

In the Euler diagram shown in Figure 2, 198 (41.3%), 230 (48.0%), and 135 (28.2%) patients had disorders in the domains of overweight/obesity, metabolic risk abnormalities, and DM, respectively. Furthermore, there was a significant overlap in the three metabolic conditions, and the most common combination involved all metabolic disorders (24.0%). The baseline characteristics were stratified by the presence or absence of MAFLD. MAFLD was associated with younger age, male sex, higher BMI, indicators of obesity, and higher liver function tests (such as aspartate aminotransferase (AST), alanine aminotransferase (ALT), and γ-GTP). 

Table 2 summarizes the multivariate linear regression analysis results used to assess the association between physical function (leg strength, gait speed, and 6 MWD) and MAFLD.

**Figure 3:**
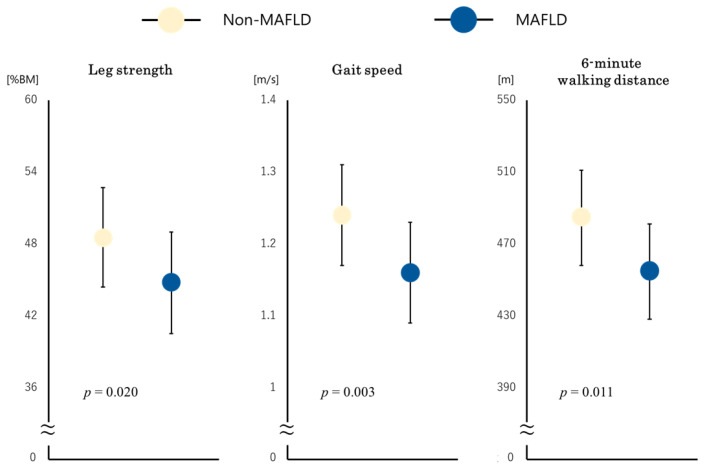
Multiple regression models of MAFLD with leg strength, gait speed, and 6 MWD. Estimated mean values of leg strength, gait speed, and 6 MWD in multiple regression models were adjusted for age, sex, BMI, log maximum CK, number of diseased vessels, prior heart failure, hypertension, dyslipidemia, diabetes mellitus, smoking, log albumin, and log hemoglobin. MAFLD, metabolic dysfunction-associated fatty liver disease; 6 MWD, 6-min walking distance; BMI, body mass index; CK, creatine kinase.

MAFLD was associated with a decline in each physical function even after adjustment for the covariates related to the severity of ACS and poor physical function (leg strength, β: −0.122, *p* = 0.020, gait speed, β: −0.159, *p* = 0.003 and 6 MWD, β: −0.114, *p* = 0.011) (Figure 3).

**Figure 4:**
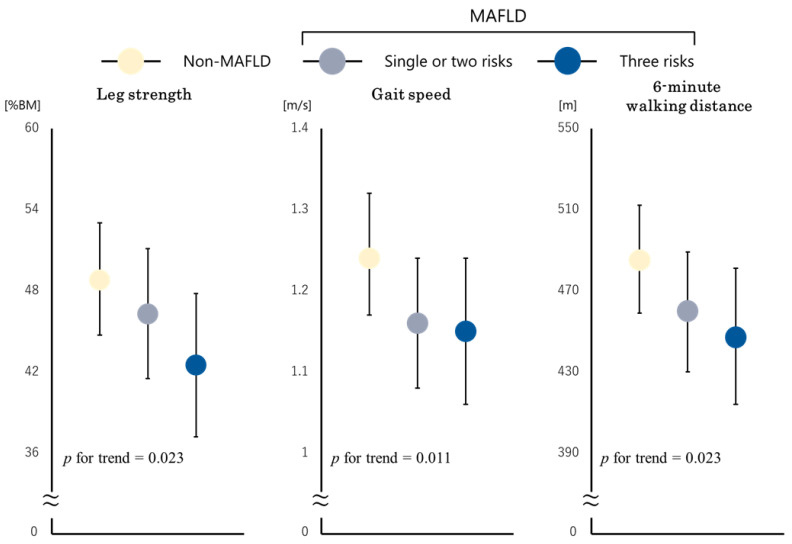
Multiple regression models of multiple metabolic condition disorders with leg strength, gait speed, and 6 MWD. The estimated mean values of leg strength, gait speed, and 6 MWD in the multiple regression models were adjusted for age, sex, BMI, log maximum CK, number of diseased vessels, prior heart failure, hypertension, dyslipidemia, diabetes mellitus, smoking, log albumin, and log hemoglobin. MAFLD, metabolic dysfunction-associated fatty liver disease; 6 MWD, 6-min walking distance; BMI, body mass index; CK, creatine kinase.

We also conducted the same analyses to evaluate the effects of multiple metabolic disorders on physical dysfunction (Figure 4). After adjusting for the same covariates, increased metabolic condition impairments in MAFLD patients were still associated with lower physical function decline (leg strength, *p* for trend = 0.023, gait speed, *p* for trend = 0.011, and 6 MWD, *p* for trend = 0.023). 

During a median follow-up period of 1.43 (IQR, 0.78–4.06) years, a total of 86 (18.0%) events were observed. The Poisson regression models were performed to evaluate the association of MAFLD and physical dysfunction and the risk of all-cause mortality and CVD re-hospitalization in patients with ACS (Table 3). The Poisson regression analysis showed that the adjusted IRRs of the non-MAFLD/low leg strength, MAFLD/high leg strength, and MAFDL/low leg strength groups compared with the non-MAFLD/high leg strength group after adjusting for age, sex, BMI, log maximum CK, number of diseased vessels, prior heart failure, hypertension, dyslipidemia, diabetes mellitus, smoking, log albumin, and log hemoglobin were 1.029 (95% CI: 0.463–2.287, *p* = 0.945), 2.390 (95% CI: 1.310–4.362, *p* = 0. 005), and 1.600 (95% CI: 0.659–3.884, *p* = 0.298), respectively. The adjusted IRRs for the non-MAFLD/low gait speed, MAFLD/high gait speed, and MAFDL/low gait speed groups compared with the non-MAFLD/high gait speed group after adjusting for the effects of the same variables were 1.665 (95% CI: 0.780–3.553, *p* = 0.187), 2.126 (95% CI: 1.140–3.964, *p* = 0.018), and 2.845 (95% CI: 1.256–6.444, *p* = 0.012), respectively. In addition, the adjusted IRRs for the non-MAFLD/low 6 MWD, MAFLD/high 6 MWD, and MAFDL/low 6 MWD groups compared with the non-MAFLD/high 6 MWD group after adjusting for the effects of the same variables were 2.116 (95% CI: 1.021–4.387, *p* = 0.044), 2.081 (95% CI: 1.071–4.040, *p* = 0.031), and 3.668 (95% CI: 1.673–8.038, *p* = 0.001), respectively.

## 4. Discussion

This is the first study to investigate the prevalence of MAFLD, overlapping metabolic abnormalities, and their impact on physical function in patients with ACS. The significant findings of this study are as follows: (1) among the patients with ACS, approximately half had MAFLD, (2) patients with MAFLD were more likely to be younger and had the highest proportion of the three metabolic abnormalities, (3) in patients with ACS, the presence of MAFLD was associated with poor physical function, (4) the higher the number of background metabolic abnormalities, the lower the physical functioning, and (5) MAFLD was associated with poor prognosis in patients with ACS. These findings underscored the importance of comprehensive evaluation of metabolic abnormalities and MAFLD even in younger patients with ACS.

Many studies have reported that NAFLD is more common in patients with IHD and combines many common risk factors, such as DM and obesity [2,5,6,11]. In this study, we defined MAFLD using the definition proposed by Eslam et al. [12] and showed that 48.9% of patients had MAFLD. This percentage was similar to that reported in a meta-analytic study that assessed the prevalence of MAFLD in obesity (50.7%) [26]. Therefore, the estimates obtained from our study sample may be close to its prevalence in a real-world clinical setting.

Secondly, it is generally reported that the prevalence of NAFLD/MAFLD tends to increase with age. On the contrary, several studies that have examined the prevalence of fatty liver disease, stratified by age, have demonstrated an increasing trend up to the age of 40–50 years but a lower prevalence after the age of 60 years [27,28,29]. In addition, a sharp increase in the prevalence of fatty liver disease in younger age groups, especially in Asians, has been reported [30,31]. In the present study, the prevalence of MAFLD was high in young people under 60 years of age and tended to decrease in older individuals. In addition, muscle weakness and decreased walking speed occurred at a high rate in patients with IHD and NAFLD [1,32,33], and previous studies have linked these conditions [34,35]. However, the present study is the first to report an association between MAFLD and physical dysfunction in patients with ACS. It is important to understand the relationship between these conditions since early treatment of MAFLD may lead to the prevention and treatment of sarcopenia and muscle weakness and further improvement of clinical outcomes such as cardiovascular events and death.

The association between MAFLD and physical dysfunction in patients with IHD can be attributed to several underlying mechanisms. Patients with NAFLD are often reported to develop sarcopenia; this can be attributed to the presence of insulin resistance, excess adipose tissue, and chronic low-grade inflammation in this population [1,6,36]. Hepatic steatosis and the resulting NAFLD can lead to additional chronic inflammation by secretion of inflammatory cytokines such as IL6, TNF-alpha, and leptin [37]. These inflammatory cytokines may also decrease the anabolic effect of insulin-like growth factor-1 and promote insulin resistance [1,38]. It is also known that in metabolic disorders such as obesity and diabetes mellitus, insulin resistance can lead to the accumulation of ectopic lipids in muscle cells and other organs [39], and weakened muscles due to these factors lead to decreased mitochondrial function [40]. However, skeletal muscle has been reported to be an endocrine organ that secretes myokines that regulate systemic metabolism [1]. The metabolic effects of irisin, a type of myokine, have been linked to improvements in glucose metabolism and hepatic steatosis [41], and skeletal muscle loss may exacerbate these conditions. These mechanisms have also been shown in studies of patients with cardiovascular disease [42]; therefore, MAFLD in patients with ACS may be more strongly associated with decreased muscle strength, walking speed, and exercise tolerance.

MAFLD and physical dysfunction are important factors associated with CVD events and mortality [17,43], and the accurate assessment of both in hospitalized patients with ACS is necessary. These conditions are not merely indicators reflecting disease severity but are treatable; thus, improving these conditions may significantly improve clinical outcomes. A study in patients with MAFLD showed that aerobic exercise improved liver fibrosis severity and VO_2max_ [44]. In addition, studies of patients with NAFLD reported that aerobic exercise, resistance training, and nutritional therapy significantly improved liver fibrosis [45,46,47]. Furthermore, it has been reported that patients with MAFLD who have a greater number of underlying metabolic disorders also have significantly higher severity of liver fibrosis [48,49]; it is widely known that cardiac rehabilitation of patients with CVD also improves their underlying metabolic disorders (DM, dyslipidemia, hypertension, etc.). Therefore, it is suggested that appropriate cardiac rehabilitation for patients with ACS may lead to improved MAFLD and prognosis.

In this study, FLI was used to assess hepatic steatosis. Biochemical data of TG and γ-GTP in patients are commonly measured in clinical practice, and WC and body weight are easily measurable, making it easy to assess hepatic steatosis in any patient. Therefore, it may be possible to evaluate MAFLD early in patients who do not have a history of liver disease and thus, do not have a detailed liver function examination. In the present study, about half of the patients with ACS had MAFLD, which was associated with decreased physical function. Our findings suggest that assessing MAFLD in patients with ACS may help detect an early decline in muscle strength and gait speed, allowing for more individualized decisions regarding therapeutic intervention and prevention of CVD recurrence in ACS patients.

## 5. Study Limitations

This study had some limitations. First, because this was a single-center retrospective observational study, the causal relationship between MAFLD and motor dysfunction could not be evaluated. Furthermore, the study did not show an association between MAFLD and the prognosis of patients with IHD. Second, this study included only Asian patients with ACS. Therefore, further studies are required to determine whether the results can be generalized to other ethnic groups. Third, approximately 80% of the subjects in this study were male patients. Therefore, the differences in the association of MAFLD with physical function may be due to the differences in gender. Fourth, in this study, hepatic steatosis was assessed using the FLI, and no additional information was obtained using liver biopsy, ultrasonography, or imaging to determine the status of hepatic steatosis. Therefore, information on liver steatosis unrelated to the severity of cardiac diseases was unknown. However, the FLI can assess fatty liver by ultrasonography with high accuracy [4,20,50] and has been reported to be associated with NAFLD development [51]. Furthermore, the Asian Pacific Association’s guidelines for the management of MAFLD recommend using FLI to evaluate hepatic steatosis [52]. Therefore, evaluation with FLI, which is easy to perform, may help in the early detection of MAFLD. Finally, there is limited information on DM and treatment status related to obesity and metabolic disorders in the context of MAFLD. Hence, there is a need to further investigate the long-term disease status and changes in physical function in MAFLD.

## 6. Conclusions

In conclusion, MAFLD is common in patients with ACS, and most patients with MAFLD have several overlapping metabolic abnormalities. MAFLD is associated with impaired physical function, and the greater the number of overlapping metabolic abnormalities, the worse the motor function. Furthermore, the combination of MAFLD and poor physical function was associated with poor prognosis in patients with ACS. Although the causal relationship between MAFLD and physical function was not determined, the assessment and intervention of the newly defined MAFLD in patients with ACS may be an early predictor of physical functional decline and may improve the prognosis and QOL.

## Figures and Tables

**Table 1:** Patient characteristics.

	Overall	Non-MAFLD	MAFLD	
	*n* = 479	*n* = 245; 51.1%	*n* = 234; 48.9%	*p*-Value
Age (years)	65 (56–73)	69 (61–74)	63 (52–70)	<0.001
Male, *n* (%)	387 (80.8)	205 (83.7)	182 (77.8)	0.128
BMI (kg/m^2^)	23.4 (21.5–25.7)	21.8 (20.3–23.0)	25.5 (23.9–27.7)	<0.001
Heart rate (beats/min)	72 (65–84)	71 (64–82)	75 (65–86)	0.049
Systolic blood pressure (mm Hg)	114 (101–133)	113 (100–130)	117 (103–135)	0.068
Diastolic blood pressure (mm Hg)	66 (59–77)	65 (58–74)	68 (61–80)	0.001
Diagnosis, *n* (%)				
Myocardial infarction	438 (91.4)	226 (92.2)	212 (90.6)	0.631
Unstable anginaa	41 (8.6)	19 (7.8)	22 (9.4)	0.631
Treatment, *n* (%)				
PCI	412 (86.0)	209 (85.3)	203 (86.8)	0.746
CABG	32 (6.7)	19 (7.8)	13 (5.6)	0.435
Number of diseased vessels, *n* (%)				0.252
1	204 (47.1)	97 (43.9)	107 (50.5)	
2	147 (33.9)	83 (37.6)	64 (30.2)	
3	82 (18.9)	41 (18.6)	41 (19.3)	
LVEF (%)	53.0 (45.0–60.0)	52.0 (44.5–61.2)	53.6 (45.0–60.0)	0.920
Prior heart failure, *n* (%)	26 (5.4)	16 (6.5)	10 (4.3)	0.374
Waist circumference (cm)	87.0 (81.5–93.0)	82.5 (78.0–87.0)	92.5 (87.8–97.5)	<0.001
Comorbidities				
Hypertension, *n* (%)	375 (78.3)	183 (74.7)	192 (82.1)	0.066
Dyslipidemia, *n* (%)	539 (68.1)	251 (60.2)	288 (76.8)	<0.001
Diabetes mellitus, *n* (%)	256 (53.4)	123 (50.2)	133 (56.8)	0.173
Obesity, *n* (%)	158 (33.0)	18 (7.3)	140 (59.8)	<0.001
Current smoker, *n* (%)	178 (38.0)	82 (34.2)	96 (41.9)	0.102
Medications				
Beta Blocker, *n* (%)	423 (88.3)	214 (87.3)	209 (89.3)	0.597
ACE inhibitor or ARB, *n* (%)	433 (90.4)	222 (90.6)	211 (90.2)	0.993
Statin, *n* (%)	451 (94.2)	236 (96.3)	215 (91.9)	0.060
Aspirin, *n* (%)	319 (66.6)	163 (66.5)	156 (66.7)	0.999
Laboratory examination				
CRP (mg/dL)	0.40 (0.10–1.10)	0.40 (0.10–1.00)	0.40 (0.20–1.20)	0.639
Triglyceride (mg/dL)	114 (89–149)	98 (78–123)	139 (107–174)	<0.001
Total cholesterol (mg/dL)	143 (126–163)	137 (123–159)	146 (132–167)	0.003
LDL-C (mg/dL)	82 (67–98)	78 (65–97)	84 (72–100)	0.006
HDL-C (mg/dL)	39 (33–46)	41 (34–50)	37 (32–43)	<0.001
Total bilirubin (mg/dL)	0.5 (0.4–0.7)	0.5 (0.4–0.6)	0.5 (0.4–0.7)	0.006
AST (U/L)	22 (17–29)	21 (17–27)	22 (18–31)	<0.001
ALT (U/L)	24 (16–37)	21 (15–31)	28 (20–45)	<0.001
γ-GTP (U/L)	34 (23–53)	27 (20–42)	45 (29–82)	<0.001
Albumin (g/dL)	3.8 (3.4–4.1)	3.7 (3.3–4.0)	3.9 (3.5–4.1)	0.002
Hemoglobin (g/dL)	13.1 (12.0–14.3)	12.8 (11.7–14.1)	13.4 (12.3–14.6)	<0.001
HbA1c (%)	6.0 (5.5–6.7)	5.8 (5.4–6.5)	6.1 (5.6–6.8)	<0.001
Maximum CK (U/L)	2122 (1014–3839)	2041 (952–3683)	2279 (1075–4198)	0.238
Fatty liver index (point)	33.0 (17.4–54.2)	17.7 (11.5–26.6)	55.1 (42.8–68.9)	<0.001
Physical function				
Leg strength (%BM)	47.4 (37.1–61.7)	47.6 (37.8–63.5)	47.0 (37.0–58.9)	0.226
Gait speed (m/s)	1.2 (1.0–1.4)	1.2 (1.1–1.4)	1.2 (1.0–1.3)	0.891
6-min walking distance (m)	486 (405–544)	483 (405–540)	489 (406–550)	0.534

Median (interquartile range); *n*, number (%); MAFLD, metabolic dysfunction-associated fatty liver disease; BMI, body mass index; PCI, percutaneous coronary intervention; CABG coronary aortic bypass graft; LVEF, left ventricular ejection fraction; ACE, angiotensin-converting enzyme; ARB, angiotensin receptor blocker; CRP, C-reactive protein; LDL-C, low-density lipoprotein cholesterol; HDL-C, high-density lipoprotein cholesterol; AST, aspartate aminotransferase; ALT, alanine aminotransferase; γ-GTP, gamma-glutamyl transpeptidase; HbA1c, hemoglobinA1c, CK, creatine kinase.

**Table 2:** Associations of metabolic dysfunction-associated fatty liver disease with physical function tests.

	Leg Strength				Gait Speed				6-Min Walking Distance			
Effect	B Coefficient	β	t Value	*p*-Value	B Coefficient	β	t Value	*p*-Value	B Coefficient	β	t Value	*p*-Value
MAFLD	−3.996	−0.122	−2.327	0.020	−0.089	−0.159	−3.021	0.003	−28.986	−0.114	−2.557	0.011
Age	−0.444	−0.304	−6.428	<0.001	−0.008	−0.323	−6.548	<0.001	−4.112	−0.410	−9.388	<0.001
Sex (male)	11.31	0.258	6.200	<0.001	0.088	0.121	2.729	0.007	63.416	0.207	5.125	<0.001
BMI	−0.427	−0.082	−1.655	0.099	0.007	0.088	1.664	0.097	0.886	0.022	0.536	0.592
Log maximum CK	0.093	0.014	0.129	0.897	0.000	−0.006	0.006	0.995	−1.899	−0.017	−0.398	0.691
Number of diseased vessels	−0.385	−0.007	−0.427	0.670	−0.023	−0.058	−0.673	0.165	−15.263	−0.091	−0.398	0.691
Prior heart failure	−0.603	−0.008	−0.210	0.834	−0.019	−0.020	−0.391	0.696	15.065	0.021	0.790	0.691
Hypertension	−2.100	−0.050	−1.269	0.205	0.009	0.012	0.296	0.768	−6.411	−0.018	−0.598	0.550
Dyslipidemia	2.632	0.071	1.767	0.078	−0.002	−0.012	−0.061	0.952	−2.367	−0.015	−0.248	0.804
Diabetes mellitus	−3.239	−0.096	−2.382	0.018	−0.016	−0.026	−0.711	0.477	−18.021	−0.073	−2.084	0.038
Smoking	−0.847	−0.021	−0.566	0.572	−0.018	−0.034	−0.712	0.477	−8.702	−0.033	−0.915	0.361
Log Alb	18.624	0.159	3.334	<0.001	0.363	0.174	3.798	<0.001	213.523	0.245	6.054	<0.001
Log Hb	17.282	0.138	2.901	0.004	0.312	0.155	3.094	0.002	107.103	0.127	2.791	0.006

β, standardized regression coefficient; MAFLD, metabolic dysfunction-associated fatty liver disease; BMI, body mass index; CK, creatine kinase; Alb, albumin; Hb, hemoglobin.

**Table 3:** Adjusted incidence rate ratio for the all-cause mortality and cardiovascular re-hospitalization events.

	Number of Events (%)	IRR	95% CI	*p*-Value
All-cause death and cardiovascular re-hospitalization	86 (18.0)			
Non-MAFLD/high leg strength		1.000	(Reference)	
Non-MAFLD/low leg strength		1.029	0.463–2.287	0.945
MAFLD/high leg strength		2.390	1.310–4.362	0.005
MAFLD/low leg strength		1.600	0.659–3.884	0.298
Non-MAFLD/high gait speed		1.000	(Reference)	
Non-MAFLD/low gait speed		1.665	0.780–3.553	0.187
MAFLD/high gait speed		2.126	1.140–3.964	0.018
MAFLD/low gait speed		2.845	1.256–6.444	0.012
Non-MAFLD/high 6 MWD		1.000	(Reference)	
Non-MAFLD/low 6 MWD		2.116	1.021–4.387	0.044
MAFLD/high 6 MWD		2.081	1.071–4.040	0.031
MAFLD/low 6 MWD		3.668	1.673–8.038	0.001

Adjusted by age, sex, BMI, log maximum CK, number of diseased vessels, prior heart failure, hypertension, dyslipidemia, diabetes mellitus, smoking, log albumin, log hemoglobin. IRR, incidence rate ratio; CI, confidence interval; MAFLD, metabolic dysfunction-associated fatty liver disease, 6 MWD, 6-min walking distance; BMI, body mass index; CK, creatine kinase.

## Data Availability

The datasets used and/or analysed during the current study are available from the corresponding author on reasonable request.

## Supplementary Materials

The following supporting information can be downloaded at: https://www.mdpi.com/article/10.3390/jcm11071847/s1, Figure S1: Prevalence of metabolic dysfunction-associated fatty liver disease by diabetes.
