# Supplementary figures and images for: The Prevalence of Metabolic Dysfunction-Associated Fatty Liver Disease and Its Association with Physical Function and Prognosis in Patients with Acute Coronary Syndrome
Source: J Clin Med. 2022 Mar 26;11(7):1847. doi: 10.3390/jcm11071847 (PMC8999802; doi:10.3390/jcm11071847)

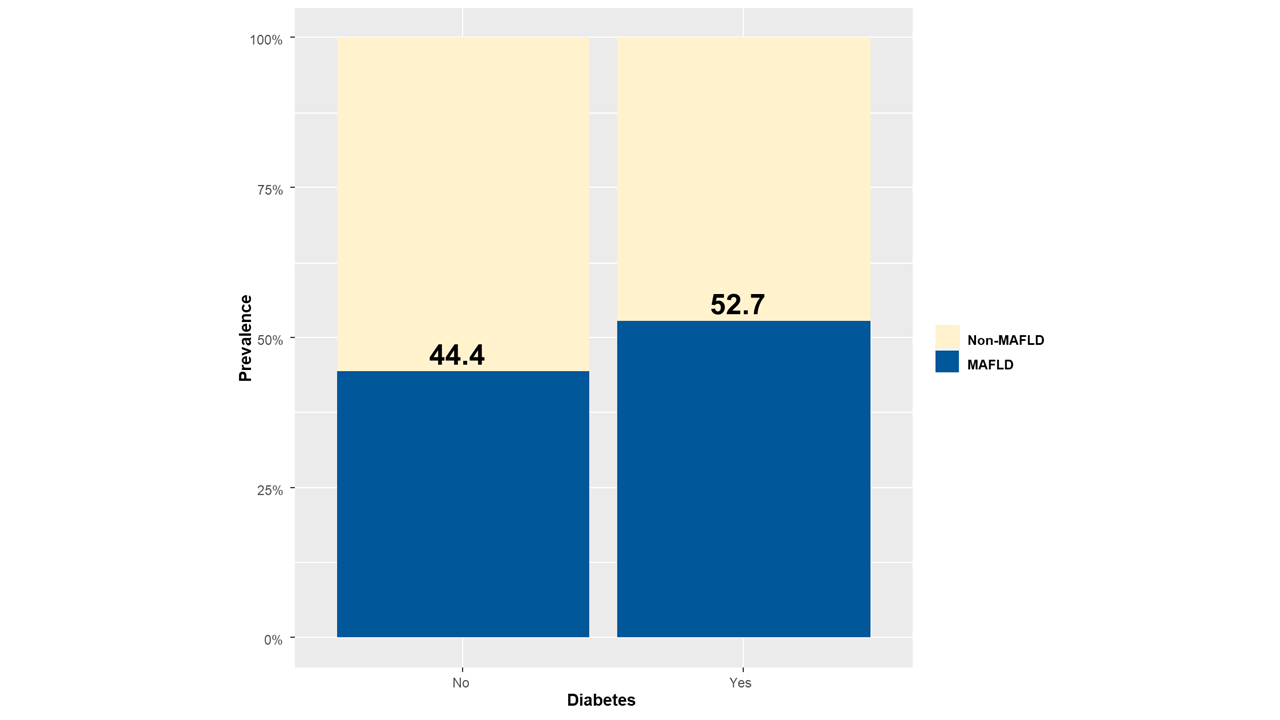

Supplement: Supplementary file 1 [file jcm-11-01847-s001.zip › supplementary-Figure S1.tif]
